# Brain benefits of deep learning-based noise management in experienced hearing aid users using functional near infrared spectroscopy

**DOI:** 10.1038/s41598-025-25801-y

**Published:** 2025-11-25

**Authors:** Jonathan M. Vaisberg, Carmen Dang, Yan Jiang, Jinyu Qian, Frank A. Russo

**Affiliations:** 1 Sonova Canada Inc., 20 Beasley Drive, Kitchener, ON N2E 1Y6 Canada; 2 Department of Psychology, Toronto Metropolitan University, Toronto, ON Canada; 3 Health & Rehabilitation Sciences Program, Western University, London, ON Canada; 4 Department of Communicative Disorders and Sciences, The State University of New York at Buffalo, Buffalo, NY USA; 5 Department of Speech-Language Pathology, University of Toronto, Toronto, ON Canada

**Keywords:** Auditory system, Cognitive neuroscience, Near-infrared spectroscopy

## Abstract

There is growing interest in using neuroimaging to understanding listening effort in individuals with hearing loss, with a particular focus on how innovative hearing aid features impact listening effort. This study used functional near infrared spectroscopy (fNIRS) to investigate the neural basis of listening effort in experienced hearing aid users. This study evaluated the impact of a new deep learning-based noise management hearing aid feature on cerebral blood oxygenation, with the expectation that it would be associated with less oxygenation in the left prefrontal cortex compared to a standard quiet-listening hearing aid program. Twenty-six experienced hearing aid users repeated sentence-final words from sentences presented in noise while wearing individually prescribed hearing aids in two conditions: a standard-listening program with an omnidirectional microphone setting and a DNN-listening program combining directional microphones with a deep-neural-network-based noise management algorithm. Listening accuracy, subjective listening effort ratings, and prefrontal oxygenation via fNIRS were measured throughout testing. As expected, the DNN-listening program was associated with higher listening accuracy, lower subjective listening effort ratings, and lower fNIRS-measured oxygenation in the left prefrontal cortex relative to the standard-listening program. The utility of fNIRS for hearing aid research and the interaction of listening effort and other cognitive processes are discussed further.

## Introduction

Listening effort, often described as the cognitive resource allocation required to understand auditory information especially under challenging listening conditions such as in speech-in-noise^1,2^, is considered an important measure for profiling hearing performance beyond more traditional measures like pure-tone audiometry or speech intelligibility. Historically, researchers have applied a wide range of subjective, objective and physiological listening effort measurement methods across diverse stimuli presentation conditions and tasks. However, these diverse studies have led to mixed and sometimes conflicting results across studies^1,3^. Central physiological measures, such as functional magnetic resonance imaging (fMRI) and electroencephalography (EEG), are a group of methods used to index changes in neural activity in response to changes in stimulus demands and can be advantageous by providing objective, real-time insight into neural activity without relying on subjective reports or behavioural performance. However, even central physiological measures are not without limitations. fMRI produces noise well above 100 dB SPL^4^ and prohibits metal objects such as hearing aids near the scanner. EEG does not share the same prohibition but warrants attention to the interaction of electromagnetic activity between external electronic devices, such as hearing aids, and EEG systems^5^. In addition, EEG has relatively poor spatial acuity, barring the ability to localize neural activity to specific regions.

These limitations have contributed to growing interest in functional near infrared spectroscopy (fNIRS) as a useful neuroimaging tool for listening effort research. fNIRS is a non-invasive, portable, and comparatively low-cost method that measures hemodynamic responses in the cortex using near infrared light^6^. fNIRS is quiet enough to allow playback of soft-level stimuli, can be worn comfortably with hearing aids, and requires relatively minimal setup. Although its spatial resolution does not match that of fMRI, fNIRS provides substantially improved spatial localization compared to EEG, enabling researchers to assess regional activation patterns in the cortex. These practical and spatial advantages make fNIRS a valuable tool for studying listening effort in the brain, particularly in study designs where techniques like fMRI and EEG are less feasible.

To use fNIRS effectively for listening effort research, background knowledge of neural sources associated with language and listening effort is essential. The “core” language network is generally localized to left-hemisphere frontal and temporal areas in the brain^7^, and a meta-analysis of neuroimaging studies identifying cortical activation patterns while participants underwent different speech-in-noise tasks supports this^8^. Generally, the right insula, left inferior parietal lobule and left inferior frontal gyrus are most active during more adverse listening conditions, with the left inferior frontal gyrus showing the largest activation^9–13^. The inferior frontal gyrus is associated with speech production, comprehension, and cognitive control especially under challenging listening conditions^14^ while the inferior parietal lobule serves to integrate acoustic and linguistic representations of said speech^15,16^.

Neuroimaging methods are particularly valuable for studying hearing aid usage, as they offer relatively more objective data that may provide complementary insights into the behavioural benefits of wearing hearing aids. Rovetti et al^17^. studied the prefrontal cortex using fNIRS in hearing aid users during an auditory n-back task. Listeners were required to memorize and recall auditory digit sequences of varying lengths in aided and unaided conditions. While increasing the sequence length was associated with increased prefrontal activation, interpreted as increased listening effort, no differences were found between aided and unaided listening. The outcome may have been limited in that hearing aid fittings were not controlled across participants, nor was it clear how the n-back task represented real-world communication. Vaisberg et al^18^. also used fNIRS to study the activation of prefrontal cortex in experienced hearing aid users. In contrast to Rovetti et al^17^., this study directly targeted listening effort by employing a task more ecologically valid and closely aligned with real-world speech processing demands, rather than relying on a more abstract working memory paradigm such as the n-back task. Participants were instructed to repeat sentences at hard and easy signal-to-noise ratios (SNRs), both with and without hearing aids while fNIRS was measured throughout. Easier listening conditions (better SNR and use of hearing aids) were associated with improved listening accuracy, reduced subjective listening effort, and lower prefrontal cortex oxygenation. Listening accuracy and subjective effort were also significant predictors of cerebral blood oxygenation, suggesting that easier listening conditions required less listening effort, thereby potentially freeing up cognitive resources for other tasks. To our knowledge, this continues to be the only study to date concerning fNIRS and hearing aids in a speech understanding task. Despite broadening our understanding of hearing aid benefits, the latter study’s most impactful outcome was revealing fNIRS as a tool sensitive to aided versus unaided listening, since decades of behavioural and real-world evidence already establish that hearing aids improve communication for people with hearing loss.

Having gathered initial evidence that fNIRS is sensitive to aided versus unaided listening in a speech understanding task, it is next important to learn whether fNIRS is sensitive to different hearing aid features. Cortical differences between hearing aid features may guide manufacturers and clinicians alike to assess whether new hearing aid features improve communication, including reducing listening effort, relative to older features. To our knowledge, no studies to date have utilized fNIRS to investigate between-feature differences. Learning whether fNIRS can do the same will establish it as a neuroimaging tool that can be used in the study of new hearing aid features.

## Current study

The current study seeks to understand whether fNIRS is sensitive to differences between hearing aid features’ impacts on cortical oxygenation and builds on our previous study, in which fNIRS was sensitive to differences between aided and unaided listening^18^. In the current study, participants were instructed to repeat the final words from Revised-Speech-In-Noise-Test (R-SPIN)^19^ sentences in rear-presented noise in a one-factor, two-level factorial design while oxygenation was measured across the prefrontal cortex. The factor levels were two programs on a commercially available hearing aid, Phonak Audéo I90-Sphere: (1) a standard (quiet listening) program comprised of omnidirectional microphones, fast acting compression, and limited noise management; and (2) a deep neural network (DNN) program that uses artificial intelligence to remove noise from speech in real time in conjunction with directional microphones forming a static directional response. The DNN uses 4.5 million parameters, trained on 22 million sound samples, to enhance performance across diverse listening environments^20,21^. The DNN processes a full-spectrum audio signal, dividing it into 64 frequency bands that include both amplitude and phase components. This method allows the DNN to more effectively separate speech from noise compared to conventional algorithms, which typically operate with lower frequency resolution and real-valued filters. By employing a complex-valued filter mask, the DNN achieves more accurate speech signal reconstruction, unlike traditional approaches that mainly reduce gain in quieter segments. This DNN technology has been associated with improvements in speech-in-noise performance compared to a quiet-listening program in listeners with severe-to-profound hearing loss^22^ and compared to a quiet-listening and a directional-microphone-alone program in listeners with moderate-to-severe hearing loss^23^.

Like our previous study, the main dependent variable was cerebral oxygen exchange (*HbDiff *= *HbO - HbR*), which captures the combined changes in oxygenated (*HbO*) and deoxygenated (*HbR*) blood, reflecting the degree of activation as oxygenated blood enters and deoxygenated blood exits a region^24^. *HbDiff* is a sensitive indicator of cerebral activation and has been used to assess cognitive effort^25,26^, including listening effort^27^. The dominance of the left inferior frontal gyrus, in prior research on listening effort positioned the left prefrontal cortex as the primary region of interest. Additional dependent measures included subjective listening effort ratings and listening accuracy and were used to complement *HbDiff*.

## Materials and methods

### Participants

**Fig. 1:**
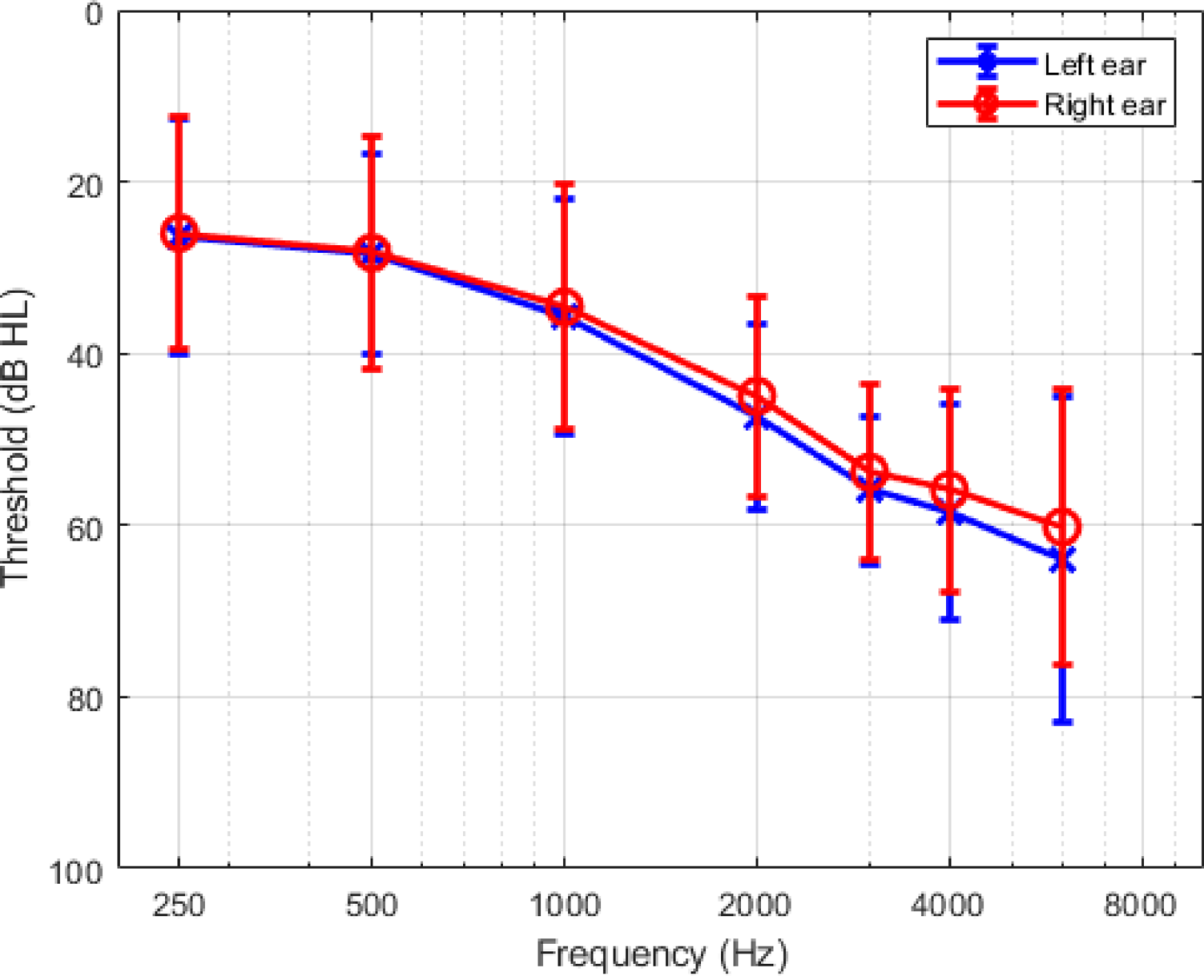
Mean air conduction thresholds for participants’ left (blue) and red (right) ears. Error bars represent one standard deviation.

Twenty-six English-speaking participants (mean age ± SD = 69.2 ± 7.1 years; 14 males, 12 females) recruited from the Sonova Audiology Research Centre - Canada research database completed the study. All participants were experienced hearing aid users (mean experience ± SD = 9.5 ± 8.5 years) and presented with an average symmetrical mild sloping to moderately-severe sensorineural hearing loss (Fig. 1). The study was approved by Western Copernicus Group Institutional Review Board and Toronto Metropolitan University Ethics Board. All participants provided written informed consent and were financially compensated for their participation. The investigation was conducted in accordance with the principles described in the Declaration of Helsinki (2018) and in compliance with ISO 14,155 (2020).

### Design

The experiment was a one-factor (two-level) repeated-measures within-subjects design. Each participant completed the experiment in a single two-hour session scheduled during standard daytime hours, which may have reduced potential effects of circadian variation on fNIRS outcomes^28^. The independent variable was hearing aid program, comprising (1) standard and (2) DNN-based programs as factor levels. The dependent variables, like our previous design^18^, were listening accuracy, subjective listening effort, and *HbDiff*. Listening accuracy was measured as percent of correctly repeated words. Listening effort was measured on a 7-point Likert scale^29^ (1-no effort, 2-very little effort, 3-little effort, 4-moderate effort, 5-considerable effort, 6-much effort, 7-extreme effort). *HbDiff* was measured using a prefrontal fNIRS system consisting of six active and two short-separation optodes positioned across the prefrontal cortex.

### Test materials

Stimuli consisted of sequences of up to five R-SPIN sentences presented in noise. Sequences, rather than individual sentences, were used to leverage the superpositioning of the response functions for each sentence in the sequence to enhance the overall temporal summation, thus increasing the SNR of the overall hemodynamic response^30–33^. Sequences varied in number of sentences so that participants would be unable to anticipate which word to repeat, forcing them to attend to the entire sequence. Sequences of three or more sentences were comprised of low-context sentences and were 18 or more seconds in duration. The shorter “decoy” sequences were comprised of one or two high-context sentences and were only included to create the impression that some sequences would contain only that number of sentences. The shorter high-context sequences’ data were not used for analysis. Noise consisted of multitalker babble and was gated on and off with each sentence. Speech was presented at individualized SNRs relative to the noise level, which was maintained at 70 dB(A).

### Equipment

#### Stimulus playback

The experiment was conducted in a double-walled, sound-attenuated booth. The participant was seated in the middle of the booth. The listening paradigm was administered using a custom MATLAB script (R2021b) running on a Windows computer located outside the booth, which controlled stimulus playback, recorded participant responses, and transmitted stimulus onset and offset markers to the fNIRS recording software. The Windows computer was connected to an RME Fireface UCX sound card, which routed the stimuli to two self-powered Genelec 8030C speakers positioned at 0° and 180° relative to the participant’s chair, each placed 1.3 m away from the participant’s head. Speech was presented from the front speaker while noise was presented from the rear speaker.

#### fNIRS device

**Fig. 2:**
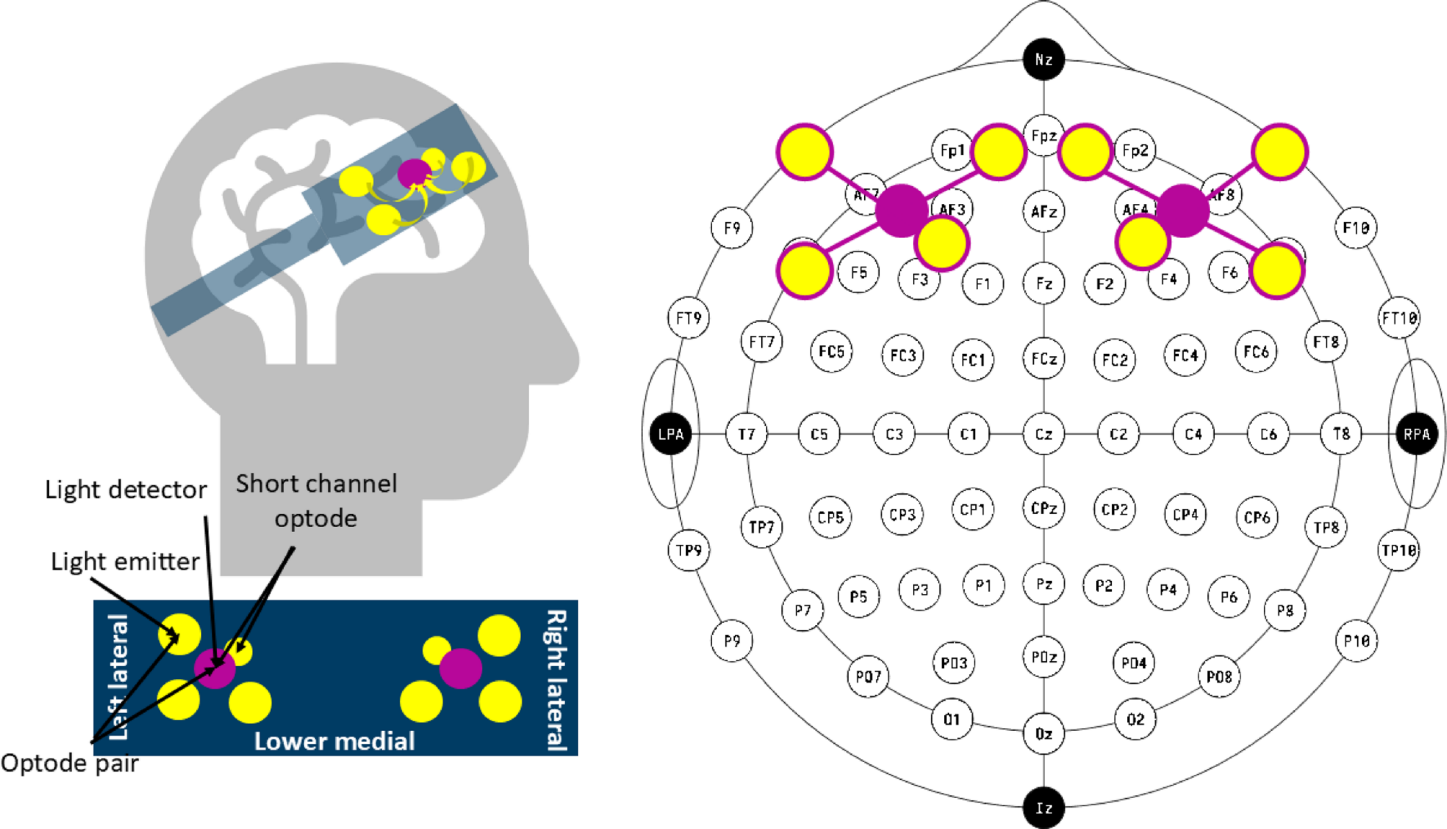
Left: Schematic of fNIRS device used in this study. Right: Eight light emitters (yellow circles) and two light detectors (magenta circles) placed over the prefrontal cortex, for a total six measurement optodes and two short-separation optodes.

Cerebral oxygenation was measured using an OctaMon continuous-wave fNIRS device (Artinis Medical Systems, Netherlands) with six active optodes and two short-separation optodes, consisting of eight LED diode emitters (wavelengths: 760 and 850 nm) and two photodiode detectors, sampled at 10 Hz. The light emitters and detectors were spaced approximately 3.5 cm apart, allowing penetration of about 1.75 cm into the cortex. The device was positioned over the participant’s prefrontal cortex. Figure 2*(*left) illustrates the fNIRS device as worn by a participant, while Fig. 2 (right) shows the optode montage overlaid on a 20-10 coordinate system. The base of the fNIRS headband was aligned just above the nasion, ensuring the lower medial light emitters were symmetrically positioned around the FpZ coordinate^34^. Recordings were transmitted via Bluetooth to the OxySoft software (version 3.2.70) on a Windows computer. The data were initially saved in *.oxy4* format and later converted to *.snirf* format for analysis using MATLAB-integrated Homer3 scripts.

#### Hearing aids

The hearing aids used were Phonak Audéo I90-Sphere receiver-in-the-canal devices and coupled to participants’ ears using occluding silicone power domes. The hearing aids were programmed using Phonak’s proprietary gain formula and fine-tuned individually to NAL-NL2 targets^35^ by a licensed audiologist. Phonak’s “Calm Situation” and “Spheric Speech in Loud Noise” programs, with all program features at default values, were used for the standard program and DNN-listening program conditions, respectively. The standard-listening program is the hearing aid’s default start-up program and implements omnidirectional microphones, speech enhancement and fast-acting compression - features common across manufacturers’ standard-listening programs. The DNN-listening program implements a combination of directional microphones, DNN-based noise management, and slow-acting compression. Hearing aids were worn for the full duration of testing.

### Procedure

#### SNR-50 search

The experiment began with an SNR-50 measurement for each participant, which is the SNR at which they could repeat 50% of the presented words. Individual SNR-50 measurements calibrated the “difficulty” of the task such that participants would perform equally despite differences in thresholds or other characteristics. Participants wore the hearing aids in the standard-listening program during the SNR-50 search procedure. This procedure followed modified Hearing-In-Noise-Test (HINT) SNR adaptation rules^36^ and the manual adaptive level controls from the Oldenburg Matrix Test^37^. In each SNR-50 run, participants listened to 20 low-context R-SPIN sentences and repeated the final word of each. The SNR was adjusted dynamically - decreasing following a correct response and increasing after an incorrect one. The noise level was maintained at 70 dB(A) and the speech level varied systematically. The starting SNR was 8 dB. For the first five sentences, the SNR was adjusted in ±4 dB steps, while the remaining sentences were adjusted in ±2 dB increments. The SNR-50 was calculated as the mean SNR from sentences 12 to 20, plus the estimated SNR after the final trial. Each run consisted of 20 sentences randomly selected from the 25 available per R-SPIN list, with sentences from only one list used per run. Participants completed a total of three runs, with the average SNR-50 plus 2 dB of the last two runs used for testing. The additional 2 dB were added to increase performance above chance so that participants would answer correctly more frequently and feel more motivated during testing. Furthermore, prior research using both subjective effort ratings and pupillometry (a physiological measure in which increased pupil dilation reflects greater cognitive demand) has shown that listening effort tends to peak when speech recognition is in the range of approximately 30% to 70%, corresponding to SNRs that are several decibels more favourable than the SNR-50 threshold^38,39^. Therefore, adding 2 dB to each participant’s SNR-50 may have not only improved motivation and performance, but also sustained cortical activation near the point of peak listening effort. Throughout the task, the experimenter recorded participants’ responses in real time to assess accuracy and trigger the next sentence. Across participants, the average SNR-50 was −0.6 dB (SD = 3.9).

#### fNIRS recording

Following the SNR-50 measurement, the experimenter placed the fNIRS device on the participant and paired it with the Oxysoft recording software. The signal quality was verified using the software’s signal quality index algorithm^40^, ensuring that the fNIRS recording SNR was above the noise floor and not saturated by ambient lighting. If the signal quality was insufficient, the experimenter adjusted the device fit.

Next, the experimenter provided the instructions for the main study task to the participants. The experimenter advised participants that they would hear sequences of sentences. Participants were instructed to repeat the last word of the last sentence in the sequence and then provide the number corresponding to their average listening effort rating for all the sentences in the sequence. Participants were informed that different hearing aid programs would be used during testing but were not told how the programs were expected to influence listening performance, nor were they informed when each program would be presented. Participants were then told that there would be a 30-second rest period between sequences and were asked to minimize movement and mind-wandering during baseline periods and testing. The experimenter was able to postpone the start of the following sequence (therefore elongating the baseline recording) in case the participant moved during the rest period.

The study task consisted of testing blocks, with each block containing a total of eight events or sentence sequences: six sequences of three or more low-context sentences and two “decoy” sequences of one or two high-context sentences. As noted in the instructions provided to participants, each sequence was separated by a 30-second silent baseline recording period. Each block comprised sentences taken from a single R-SPIN list not used in the SNR-50 task. Altogether, participants completed five blocks: a practice block and four test blocks. The practice block was completed using the standard-listening program and two of the remaining four test blocks were assigned to either the standard- or DNN-listening conditions, for a total of 16 sequences per condition. Test block order and R-SPIN list-block assignment was randomized between participants.

### Analysis

#### fNIRS data processing

The fNIRS processing pipeline resembled our previous study’s pipeline^18^, which was adapted from Zhou et al.^41^, and was entirely implemented using MATLAB. The exception between our studies was that the current design incorporated short-separation channel subtraction. The fNIRS processing occurred as follows:

1. *Removal of step-like noise.* Brief losses of contact between the fNIRS headband and skin might produce abrupt steps in the fNIRS morphology and can negatively impact signal quality. These steps were removed by calculating the derivative of each channel’s intensity time series, and absolute values greater than two SDs over the derivative’s mean were zeroed. Channels were recalculated using the cumulative sum of the updated derivatives.
2. *Exclude channels of poor quality.* Signal quality was assessed using the scalp coupling index^42^ (SCI). The SCI extracts the heartbeat from the two fNIRS wavelengths by bandpass filtering between 0.5 and 1.5 Hz and then correlating the two filtered wavelengths. A poor correlation suggests that the fNIRS measurements did not accurately capture the heartbeat and would therefore not contain other meaningful physiological responses. Channels with an SCI < 0.75 were rejected, resulting in 11.5% of channels being rejected across participants.
3. *Conversion of light intensity to optical density.*Refer to Hupper et al.^43^.
4. *Correction of motion artifacts.* Movements during testing risk physical displacement of the optodes from the participant’s head, risking noise in the data. The wavelet decomposition method^44^ was used to correct motion artifacts for any wavelet coefficients outside an interquartile range of 0.1.
5. *Conversion of optical density to hemoglobin concentration using the Modified Beer—Lambert law.*Refer to Delpy et al.^45^.
6. *Band-pass filter (0.01–1.5 Hz).* Low-frequency noise (such as drift) and high-frequency noise (such as breathing or heart rate) was band-pass filtered from the fNIRS signal.
7. *Short-separation channel correction:* Each channel underwent short-separation channel correction such that a general linear model was fit to the channel’s fNIRS signal with the timeseries of the nearest short-separation channel (based on Euclidean distance) as the regressor. The physiological noise measured by the short-separation channel was regressed out of each channel’s output.
8. *Band-pass filter (0.01–0.09 Hz).* An additional band-pass filter was applied to the data to remove any physiological noise missed prior to short-separation channel subtraction.
9. *Averaging.* HbO and HbR were calculated for each sequence of sentences. The hemodynamic response was measured as the average of 10 s following each sequence onset to the end of each sequence minus a 5-second baseline average prior to the start of the sequence. Ten seconds was chosen between sequence onset and measurement onset to allow for the hemodynamic response to reach its peak^30,46,47^. Hemodynamic responses were then averaged across all low-context sequences for a given condition.
10. *Data for analysis.* The fNIRS response used for statistical analysis was calculated as the cerebral oxygen exchange ( = *HbO - HbR*). Three subregions, each containing averages across two optodes, were used for analysis. Lateral regions contained two peripheral optodes for each respective side and the lower medial region contained the two medial optodes.

#### Statistical modelling

Aligned with previous literature, subjective listening effort ratings and listening accuracy scores were averaged across trials resulting in a mean listening effort value and a mean accuracy score per participant. Afterwards, mean listening accuracy scores were transformed to rationalized arcsin units to reduce clustering of scores near ceiling performance^48^. To assess the effect of the hearing aid program on subjective listening effort and on listening accuracy, two paired samples t-tests were conducted. A standardized effect size was calculated for each test using Cohen’s *d*.

Blood oxygenation data (*HbDiff*) were modelled using multilevel modeling (MLM) because of the clustered data structure (i.e., high levels of covariance) due to the repeated measurement design. MLM estimates random effects for each cluster to provide unbiased model estimates (standard errors, regression coefficients, *p*-values)^49^. Furthermore, MLM is robust against cases of missing data, such as rejected channels falling below the SCI threshold^50^. In contrast, an Analysis of Variance (ANOVA) is a complete-case analysis where an entire participant’s data would be removed if the participant had any missing data from rejected channels.

We conducted three MLMs with *HbDiff* as the outcome variable: an unconditional model and two a priori theory-driven models to assess the effect of the hearing aid program and whether the effect of the hearing aid program is moderated by subregions of the prefrontal cortex. The main predictor variable was the hearing aid program (standard vs. DNN) and an interaction term between the hearing aid program and prefrontal subregion (left lateral vs. lower medial vs. right lateral). Handedness was included as a covariate due to the established association between handedness and lateralization of language-dominant areas in the brain^51^, with left-handed individuals exhibiting more bilateral language processing resulting in attenuated left-hemisphere activation compared to their right-handed counterparts. Categorical variables (hearing aid program, prefrontal subregion, and handedness) were dummy-coded. The standard-listening and the left-lateral subregion were used as the reference group for the hearing aid program and prefrontal subregion, respectively. Left-handedness was the reference group for the covariate. Simple slopes analysis was used to probe the interaction.

To assess which model best fit the data, a nested-modeling approach was used. This approach incrementally adds predictor variables to a simpler model and assesses whether the additional terms significantly improve the model’s ability to account for variance in the data. Model comparisons are evaluated using likelihood ratio tests and are considered a standard method for comparing nested models in mixed-effect designs^52^ The Akaike information criterion (*AIC*) was also used to assess model fit with lower *AIC* values indicating a better fit to the data^53^.

All analyses were conducted in R version 4.4.2. MLMs were estimated using full information maximum likelihood (FIML) and conducted with the following packages: lme4^54^, lmerTest^55^, performance^56^ and interactions^57^. Paired samples t-tests were conducted using the stats package^58^ and Cohen’s *d* was calculated using the lsr package^59^. All statistical analyses were two-tailed tests with *α* = 0.05.

## Results

### Subjective listening effort

**Fig. 3:**
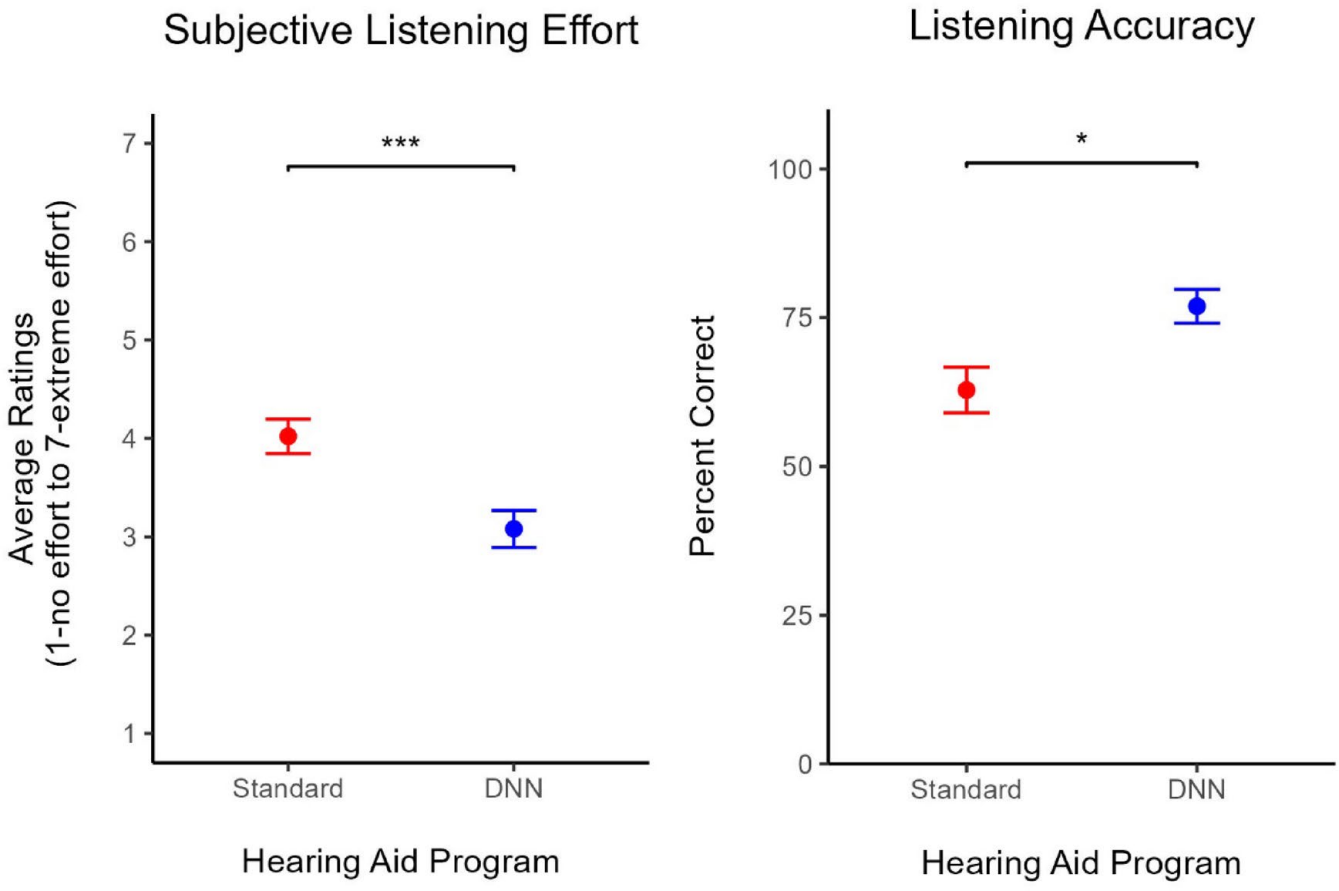
Estimated marginal mean listening effort (left) and listening accuracy (right). Error bars represent the standard error of the mean. *p <.05. **p <.01. ***p <.001. Standard refers to the hearing aid’s default start-up program, which implements omnidirectional microphones, speech enhancement and fast-acting compression. DNN refers to the DNN-listening program, which implements a combination of directional microphones DNN-based noise management, and slow-acting compression.

The hearing aid program significantly impacted listening effort such that on average, listening effort ratings were 0.94 points lower in the DNN-listening program (*M* = 3.08, *SD* = 0.96) compared to the standard-listening program (*M* = 4.02, *SD* = 0.89), *t*(25) = 10.05, *p* <.001, 95% CI [0.75, 1.13] with a Cohen’s *d* = 1.97 indicating a very large effect size^60^. Results are illustrated in Fig. 3 (left) and listed in Table 1.

**Table 1:** Paired samples T-Test for the effect of hearing aid program on subjective listening Effort.

Group	*M*	*SD*	*Diff*	*Diff* 95% CI	*df*	*t*	*p*	*d*
Standard	4.02	0.89	0.94	[0.75,1.13]	25	10.05	< 0.001	1.97
DNN	3.08	0.96						

*M* = mean; *SD* = standard deviation; *Diff* = group mean difference; *df* = degrees of freedom; *d* = Cohen’ d.

### Listening accuracy

Task accuracy scores were also significantly impacted by the hearing aid program, *t*(25) = −2.643, *p* =.013, 95% CI [−24.03, −2.98]. When participants were using the standard-listening program (*M* = 62.30, *SD* = 19.40) during the task, their accuracy scores were on average 13.50 rationalized arcsin units lower than when they were using the DNN-listening program (*M* = 75.80, *SD* = 15.20; see Fig. 3 (right) and Table 2). The effect of the hearing aid program on task accuracy corresponded to a Cohen’s *d* = 0.52, indicating a medium effect size^60^.

**Table 2:** Paired samples t-test for the effect of hearing aid program on listening accuracy. Mean values are expressed as rationalized arcsine units (*RAU*).

Group	*M*	*SD*	*Diff*	*Diff* 95% CI	*df*	*t*	*p*	*d*
Standard	62.3	19.4	−13.50	[−24.03, −2.98]	25	−2.64	0.01	0.52
DNN	75.8	15.2						

*M* = mean; *SD* = standard deviation; *Diff* = group mean difference; *df* = degrees of freedom; *d* = Cohen’ d.

### Brain

The model comparisons indicated that the model containing the interaction between hearing aid program and prefrontal subregion was a significantly better-fitting model compared to the unconditional model (*χ*^2^(4) = 19.71, *p* =.0031) and the model containing the main effect of hearing aid program alone (*χ*^2^(4) = 10.8, *p* =.029). See Table 3 for each model’s output, fit statistics and model comparison results. A simple slopes analysis was performed to probe the interaction model, and results indicated that the effect of the hearing aid program on blood oxygenation was moderated by the subregion of the prefrontal cortex.

**Table 3:** Nested model performance for the effect of condition and region on cerebral oxygen exchange (*HbO -– HbR*) Results.

	Fixed Effects					Goodness of fit and comparison			
Model	Est.	*SE*	*df*	*t*	*p*	$\:\chi\:\:$ ^2^	*AIC*	*df*	*p*
Baseline									
Intercept	0.11	2.44	26.9	0.05	0.963		20,655		
+ Main effect									
Intercept	−7.419	5.91	31.0	−1.26	0.21	8.91	20,650	2	**0.012**
Listening program (DNN)	−7.50	3.51	1775	−2.13	**0.033**				
Handedness (Right)	13.4	6.14	26.0	2.18	**0.039**				
+ Interaction									
Intercept	−7.165	6.88	55.9	−1.04	0.30	10.79	20,648	4	**0.029**
Listening program (DNN)	−18.97	6.07	1775	−3.13	**0.002**				
Region (LM)	−2.64	6.01	1783	−0.43	0.660				
Region (RL)	1.93	6.14	1795	0.31	0.753				
Handedness (Right)	134	6.17	25.9	2.18	**0.038**				
Listening program (DNN) *Region (LM)	18.42	8.49	1775	2.17	**0.030**				
Listening program (DNN) *Region (RL)	15.91	8.67	1775	1.84	0.067				

*SE* = standard error; *df* = degrees of freedom; *AIC* = Akaike information criterion; LM = lower medial; RL = right lateral. The reference groups for listening program, region, and handedness were standard listening, left lateral, and left-handedness, respectively.

**Fig. 4:**
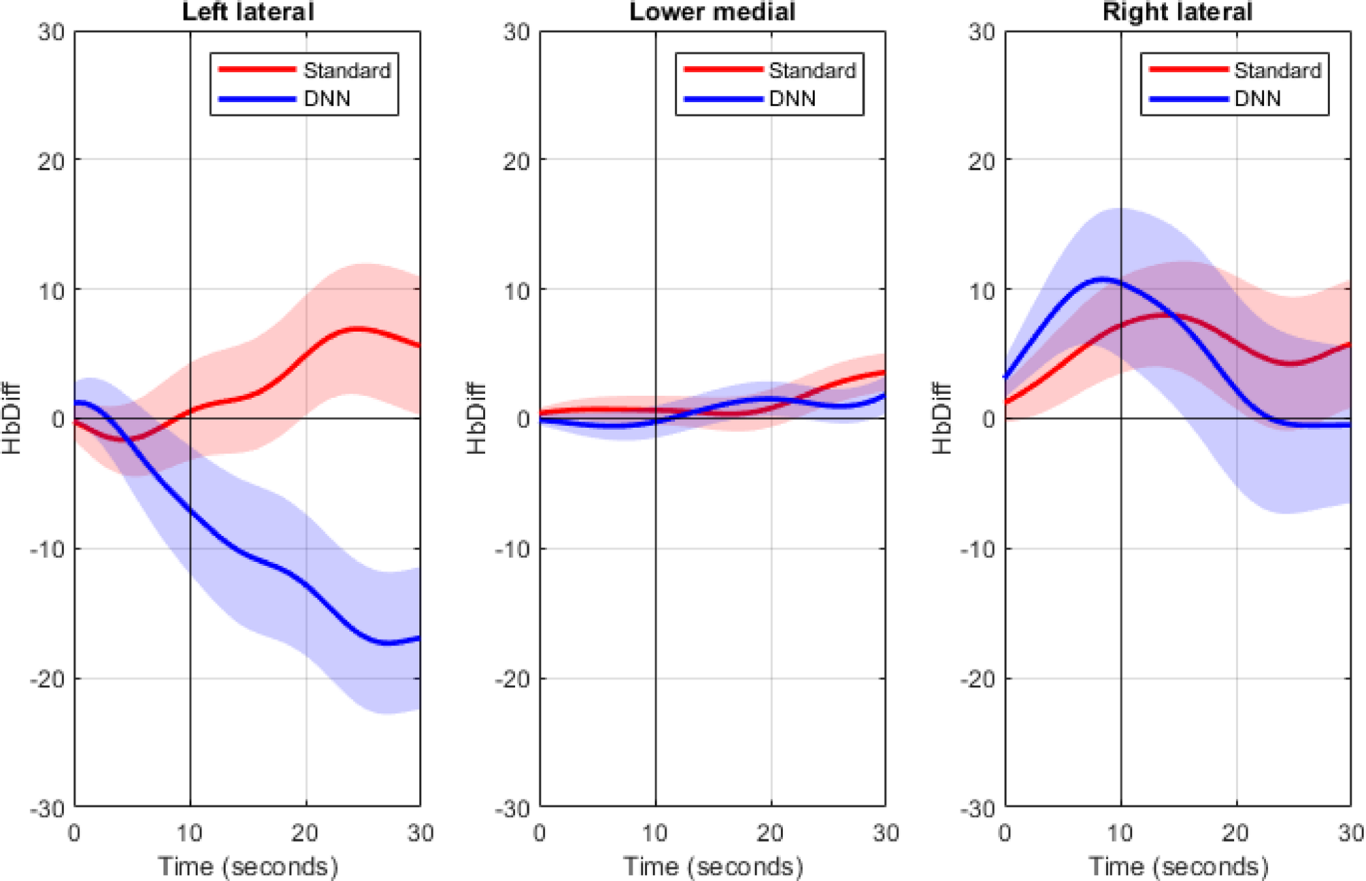
*HbDiff (HbO – HbR)* normalized time series data for each subregion averaged across participants. Shaded regions represent one standard error of the mean. Standard refers to the hearing aid’s default start-up program, which implements omnidirectional microphones, speech enhancement and fast-acting compression. DNN refers to the DNN-listening program, which implements a combination of directional microphones, DNN-based noise management, and slow-acting compression.

**Fig. 5:**
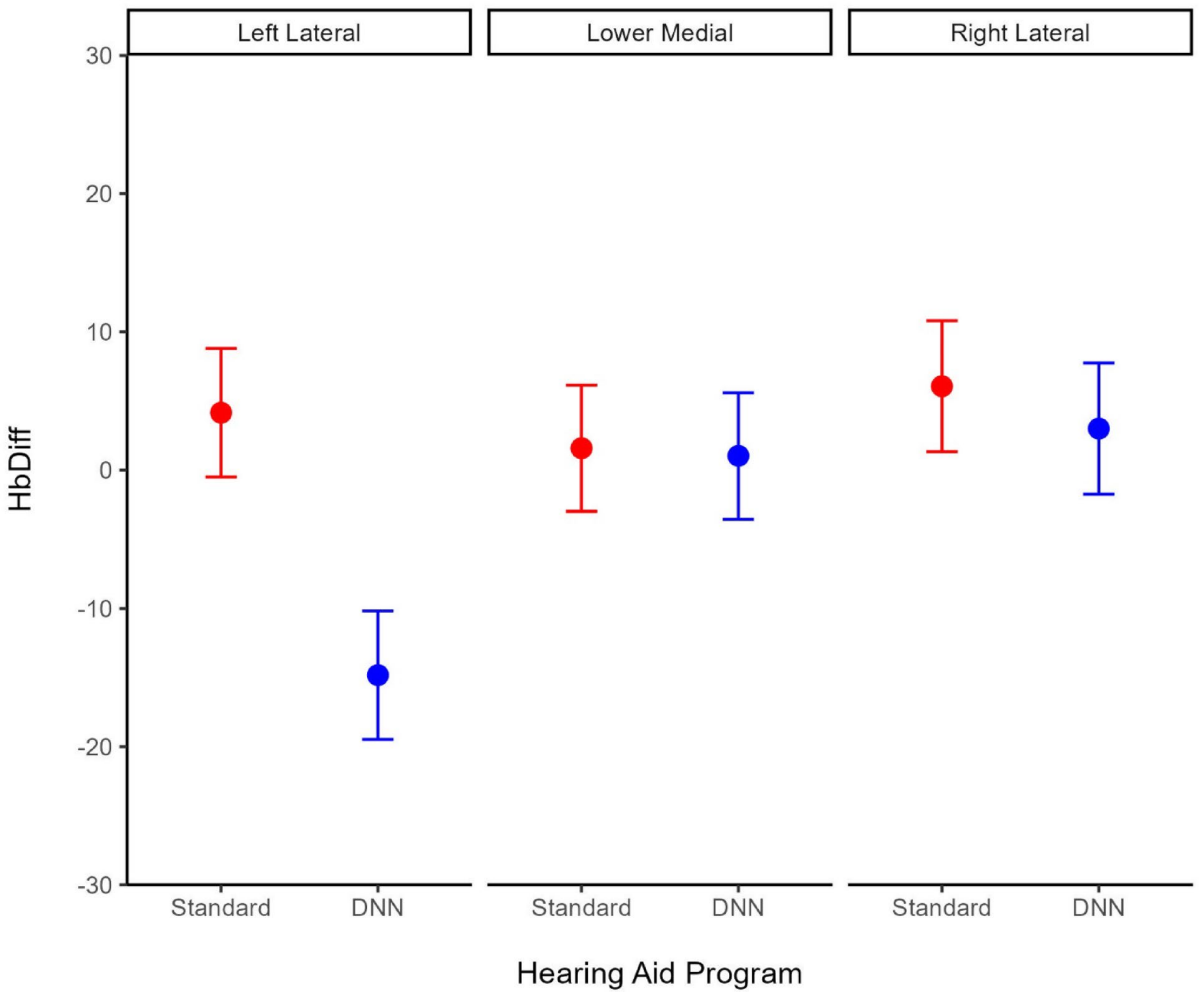
Estimated marginal mean cerebral oxygen exchange (*HbDiff; HbO* – *HbR*) by hearing aid program (Standard, DNN) for each prefrontal cortex subregion (left lateral, lower medial and right lateral) represented by a circle. Error bars represent the standard error of the mean. **p <.01. Standard refers to the hearing aid’s default start-up program, which implements omnidirectional microphones, speech enhancement and fast-acting compression. DNN refers to the DNN-listening program, which implements a combination of directional microphones DNN-based noise management, and slow-acting compression.

The hearing aid program had a significant effect on blood oxygenation in the left lateral prefrontal cortex while controlling for handedness, such that on average, blood oxygenation levels decreased by −18.97 units during the DNN-listening program, compared to the standard-listening program, *b* = −18.97, 95% CI [−30.86, −7.08], *p* =.0018. There was no effect of the hearing aid program on blood oxygenation levels in the right lateral (*p* =.62) or the lower medial (*p* =.93) prefrontal cortex. See Table 4 for summary statistics of the simple slopes analysis. See Figs. 4 and 5 for illustrations of the *HbDiff* time series and estimated marginal means, respectively.

**Table 4:** Simple slopes analysis for the interaction of condition and region on cerebral oxygen exchange (*HbO - HbR*) Results.

Region	Estimate	*SE*	95% CI	*t*	*p*
Left Lateral					
Intercept	−7.17	6.89	[−20.65, 6.33]	−1.04	0.30
Listening program	−18.97	6.07	[−7.08,−3.13]	−3.13	**0.0018**
Handedness	13.46	6.17	[1.37, 25.54]	2.18	**0.038**
Lower Medial					
Intercept	−9.81	6.85	[−26.2, 3.61]	−1.43	0.16
Listening program	−0.55	5.95	[−12.21,11.10]	−0.093	0.93
Handedness	13.46	6.17	[1.37, 25.54]	2.18	**0.038**
Right Lateral					
Intercept	−5.23	6.92	[−18.80, 8.33]	−0.76	0.45
Listening program	−3.06	6.19	[−15.20, 9.07]	−0.49	0.62
Handedness	13.46	6.17	[1.37, 25.54]	2.18	**0.038**

*SE* = standard error. The reference groups for listening program, region, and handedness were standard listening, left lateral, and left-handedness, respectively.

Handedness emerged as a significant covariate in the model hierarchy, regardless of hearing aid program or prefrontal subregion. Overall blood oxygenation levels were 13.45 units higher in right-handed participants compared to left-handed participants, *b* = 13.45, 95% CI [1.37, 25.54], *p* =.0038, reinforcing the decision to include handedness as a covariate.

### Brain-behaviour relationship

Given that an effect of listening program on blood oxygenation was found in the left lateral region only, we focused our exploratory analyses on brain-behaviour relationships involving blood oxygenation (*HbDiff*) in the left lateral prefrontal cortex. Two MLMs found that neither subjective listening effort ratings nor listening accuracy scores were significantly related to blood oxygenation. Therefore, we decided to exclude incorrect trials and analyze correct trials only, as incorrect trials might have included lapses in attention or disengagement due to task difficulty, which could have influenced the hemodynamic response in way that correct trials would not. When focusing on correct trials only, subjective listening effort was significantly related to blood oxygenation such that blood oxygenation increased by 7.89 units for every 1-unit increase on listening effort ratings, *b* = 7.89, *p* =.035. This model with blood oxygenation conditioned on listening effort was a significantly better fit to the data compared to the unconditional model (*χ²*(1) = 4.46, *p* =.035).

## Discussion

### Self-report and behaviour

This study compared impacts of a DNN-listening program, which implemented a combination of directional microphones and DNN-based noise management versus a standard-listening hearing aid program in terms of listening effort ratings, listening accuracy scores, and cerebral oxygenation. In a speech-understanding-in-noise task, experienced hearing aid users repeated an average 13.9% more words correctly and rated listening effort by an average of 0.94 fewer points while using the DNN-listening program compared to the standard-listening program. This outcome aligned with our expectations. The DNN-listening program’s directional microphones and DNN-based noise management worked in tandem to increase the functional SNR by approximately up to 9 dB^61^ at the ear relative to the standard-listening program, which used omnidirectional microphones and no DNN noise management. These findings echo those of our previous study, in which a 10 dB increase in SNR of the stimulus was associated with an average 18.9% more words repeated correctly and lower listening effort ratings by an average 0.8 points^18^ using the same sentence materials and a similar population.

Saoji et al.^22^ investigated the performance of the DNN-listening program in cochlear implant candidates, and found that the program improved speech intelligibility by 27.5% while participants repeated AzBio sentences at 5- and 10-dB SNRs. The outcomes of the current study also align with this general trend, though the observed benefits were less pronounced. Several methodological differences may account for this discrepancy. Saoji et al.^22^ had participants repeat AzBio sentences, in which participants were required to repeat entire context-rich sentences rather than low-context sentence-final words in the R-SPIN battery. Since AzBio sentences are context-rich, participants can make use of semantic cues to aid in speech recognition, potentially making it easier to repeat words in noisier conditions. In contrast, repeating only low-context words from R-SPIN sentences removes that helpful context, making the task more reliant on bottom-up acoustic cues. Therefore, low-context R-SPIN sentences have the potential to deteriorate performance compared to AzBio sentences at an equivalent SNR. However, we tested participants at individually measured SNR-50s + 2 dB using the standard-listening program, resulting in 63% correctness at an average − 0.6 dB SNR, whereas Saoji et al.^22^ tested participants at 5- and 10-dB SNRs, which were associated with an approximate 48% correctness in the standard-listening condition. In other words, the participants in Saoji et al.^22^ performed more poorly despite completing the task at easier SNRs using test materials that had additional recognition cues. A more likely methodological difference in studies explaining this discrepancy therefore relates to the audiometric profile of the study sample. Saoji et al.^22^ studied the DNN-listening program in cochlear implant candidates, who typically present with profound hearing loss, limiting their ability to benefit from conventional hearing aids. As a result, they may perform more poorly on speech recognition tasks – especially in a standard-listening program - due to severely reduced audibility, even when favourable SNRs or semantically rich sentence materials are available. They may also derive greater benefit from noise management technologies, such as DNN-listening and directional microphones, compared to listeners with milder degrees of hearing loss, such as those in the current study.

It is also important to consider that intelligibility is most sensitive to changes in SNR when baseline correctness is closer to 50%. We tested participants at individually measured SNR-50s + 2 dB using the standard-listening program, resulting in 63% correctness at an average −0.6 dB SNR, whereas Saoji et al.^22^ tested participants at 5- and 10-dB SNRs, which were associated with an approximate 48% correctness in the standard-listening condition. The latter sample may have had more to gain due to lower intelligibility in the standard-listening condition.

Finally, real-world communicative SNRs at 70 dB(A) are typically higher than those measured in the current study^62,63^ and it is possible that the DNN-listening program was developed to function optimally at more realistic SNRs than those measured here. Bench evaluations of the DNN-listening program have been shown to improve predicted intelligibility by less than 5% at −5 dB SNR, compared to 35% at 0 dB SNR, and 50% at 5 dB SNR^64^, suggesting that the benefit of the DNN-listening program is related to the SNR at which it is used. While a higher SNR in the current study may have improved DNN functionality, participants may have also scored better in the standard-listening program meaning there may have been less of an expected DNN-listening program benefit. Therefore, a trade-off had to be considered between optimal feature functionality and listening task sensitivity. More generally, the benefits of advanced signal processing, such as DNN-driven listening, may not always be captured by conventional behavioural metrics, particularly under favourable acoustic conditions where intelligibility is already near ceiling. In such contexts, physiological insights - such as those afforded by fNIRS - offer a critical means of revealing underlying differences in listening effort that may be imperceptible behaviourally. For instance, even when two behavioural outcomes appear equivalent, one score may be associated with a reduced physiological response due to DNN-listening. This study provides an important foundation for future work leveraging physiological metrics to uncover latent benefits of hearing aids, particularly under more ecologically valid SNRs where behavioural differences across programs may be minimal.

### Brain

**Fig. 6:**
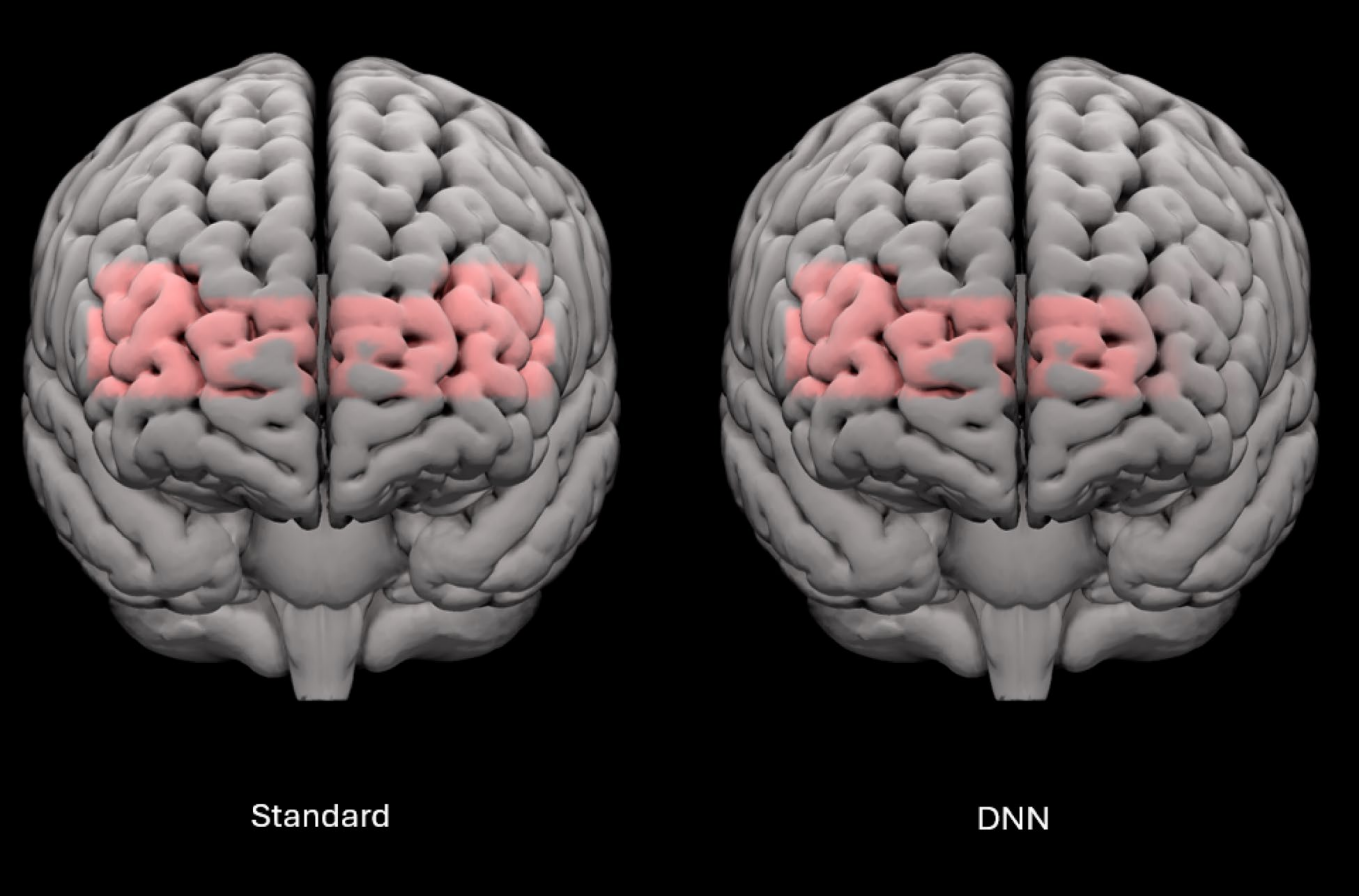
Brain activation patterns during speech-in-noise testing for the standard-listening program (left) and DNN program (right). Shading represents cortical areas of increased activation relative to baseline in standard- and DNN-listening programs. Standard refers to the hearing aid’s default start-up program, which implements omnidirectional microphones, speech enhancement and fast-acting compression. DNN refers to the DNN-listening program, which implements a combination of directional microphones, DNN-based noise management, and slow-acting compression.

The self-report and behavioural results suggest that the experiment aligned with our hypotheses. As expected, oxygenation of the prefrontal cortex as measured using fNIRS was sensitive to differences in hearing aid processing, with less oxygenation observed in the left lateral prefrontal cortex in the DNN-listening program compared to the standard-listening program (Fig. 6). These findings are consistent with and build on our previous study^18^. Recall that in that study, the objectives were to assess if (1) changes in SNR would impact oxygenation in older adult hearing aid users as it did in younger normal hearing listeners^27^ and if (2) amplification would impact oxygenation relative to unaided listening. The idea was to select conditions with the largest possible perceptual differences such that participants were as likely as possible to demonstrate differences in oxygenation. In our previous study, both SNR improvements and amplification significantly lowered oxygenation in the prefrontal cortex, interpreted as the brain requiring fewer cognitive resources at easier SNRs to complete the listening task. This outcome set the stage for between-feature comparisons, which was the current study’s objective. In this study, we selected the DNN-listening program as it combined the DNN and directional microphones to produce the greatest predicted SNR benefits when compared with other features within the hearing aid^61,64^. As hypothesized, the DNN-listening program lowered oxygenation in the prefrontal cortex by an average 18.9 µM compared to the standard-listening program. This outcome supports fNIRS as an effective tool for measuring the impact of between-feature differences on cortical oxygenation that mirrors change in subjective listening effort.

This study also adds to the growing body of literature showing associations between different hearing aid features and cortical responses. A collection of EEG studies^65–67^ demonstrated that different configurations of noise management strategies reduced listening effort during speech-understanding-in-noise-tasks, which in turn were associated with less neural activity compared to less effective noise management strategies. . However, given the poor spatial resolution of EEG, these studies were unable to localize neural activity to any specific area in the brain, and questions persist about the extent to which electromagnetic artifacts were eliminated. Our study’s DNN-listening program integrated directional microphones with DNN-based noise management, which reduced listening effort and was associated with less activity in the prefrontal cortex compared to a standard-listening program.

In the current study, differences in prefrontal cortex oxygenation were lateralized to the left side of the prefrontal cortex. A large body of research suggests that language centres and listening effort tend to be left-lateralized^7,8^, which led to the hypothesis that significant effects of oxygenation would be localized to the left side. Our previous study found that differences in prefrontal cortex oxygenation were not localized to the left lateral side, but rather across the entire prefrontal cortex^18^. While consistent with other speech-understanding-in-noise investigations in normal hearing listeners^27^, the result was not consistent with our hypothesis for that study. Bilateral activation was attributed to several reasons. Neurologically, older adults can show less hemispheric asymmetry compared to younger controls^68^, meaning they can experience greater bilateral recruitment of cortical areas for tasks that are typically lateralized. Bilateral activation might have also been observed due to methodological considerations. First, our previous study was a block-based design, in that a single hemodynamic response was measured and baseline-normalized for an entire word list. Therefore, hemodynamic responses could have been impacted by movement during speech production and/or drift due to the long duration of each hemodynamic response. In the current study, these limitations were resolved because participants were instructed to listen to a sequence of up to five sentences (lasting up to thirty seconds) while the hemodynamic response was measured, and to only respond after the sequence was finished and the measurement was finished, more closely resembling an event-based design. In doing so, we accomplished the following: participants were not speaking during fNIRS measurements, reducing the risk of motor contributions to the hemodynamic response, and multiple fNIRS measurements and baseline corrections were obtained for a single sentence list rather than only one fNIRS measurement, reducing the risk of hemodynamic drift and improving the fNIRS recording SNR per condition. Second, the lack of lateralization in our previous study could have been attributed to the lack of short-separation optodes, which are fNIRS source-detector pairs positioned at a closer distance to measure signals from extracerebral regions compared to regular optodes. These extracerebral regions include non-neural physiological processes like respiration, noncortical blood flow due to autonomic nervous system activity, and task-evoked responses or recording artefacts due to local circulatory networks supplying and draining cerebral blood flow^69^, and may have been responsible for demonstrating the effect in our previous study. Short-separation optodes are crucial for isolating and removing noise originating from extracerebral sources^6,41^, such as motion artifacts and global circulatory responses, which can mask the hemodynamic activity of interest in the cortex. By regressing out the activity captured by short-separation optodes, the contribution of noise is minimized, leading to a clearer representation of neural signals from deeper cortical regions like the left lateral prefrontal cortex.

Another interesting contrast between our studies relates to the inclusion of a practice round and its potential relationship with the absolute oxygenation values for the easiest and most difficult conditions. In our previous study, mean oxygenation in the most and least challenging conditions was approximately −20 µM and −55 µM, respectively, in the left lateral area. In the current study, mean oxygenation was 4.2 µM and −14.8 µM, respectively, which is considerably higher in magnitude. The cingulo-opercular network , which includes the dorsal anterior cingulate cortex, anterior insula, and frontal operculum, may provide an explanation. These regions, closely linked to the prefrontal cortex, are involved in top-down attentional control necessary for goal-directed behaviour, particularly in preparing for upcoming task demands^70^. The cingulo-opercular network may have been especially active during baseline recording, followed by deactivation after the stimulus onset, resulting in negative values during the event-related response. Our previous study’s procedure did not include a practice round once the fNIRS headband was already donned whereas our current procedure did include a practice round. A practice round, especially while wearing all the test equipment, helps participants familiarize with the conditions, pacing and difficulty of the task. Familiarization could have tempered the cingulo-opercular network’s involvement prior to the onset of testing, resulting in a smaller difference between the baseline and event-related responses. This may explain why the observed oxygenation values were higher in the current study compared to the previous study.

Handedness also emerged as a significant covariate in the analysis, with left-handed participants showing lower overall oxygenation levels in the prefrontal cortex compared to right-handed participants. This difference may relate to known variations in cortical organization, as left-handed individuals often exhibit more bilateral patterns of activation during language tasks^51^. However, the small number of left-handed participants in our sample (*n* = 4) relative to right-handed participants (*n* = 22) limited our ability to explore this effect further. Future work exploring the influence of handedness on speech-in-noise processing should aim to recruit a more balanced sample of left- and right-handed listeners.

#### Brain-behaviour relationship

In an exploratory analysis, we found that brain and behavioural measures were related only when participants provided correct responses during the speech task. That is, only when participants’ responses were correct did oxygenation in the left lateral prefrontal cortex decrease as participants rated less listening effort. This suggests that additional cognitive processes necessary for successful task performance, including attention, may be important for engaging the brain regions involved in speech processing. This contrasts with previous investigations where brain and behaviour were broadly associated without participants having to achieve a specific outcome^18,65–67^. A study by Wild et al^15^. supports the idea that brain/behaviour relationships may be conditional, showing that attention plays a pivotal role in understanding degraded speech. Participants were exposed to both clear and degraded speech, with brain activity recorded using fMRI before and after the study task. The study employed a dual-task paradigm, where participants either focused on the speech comprehension task alone or were distracted by an additional task to manipulate their attention levels. Results showed that when participants directed their attention to the speech task, brain regions involved in auditory processing (such as the inferior frontal gyrus) exhibited greater activation and were more sensitive to differences between conditions. These findings align with our own results, where brain and listening effort ratings were linked only when participants provided correct responses, which we posited was linked to directing attention to the task.

## Conclusion

In conclusion, this study highlights fNIRS as a useful tool for detecting cortical differences associated with different hearing aid features in a speech understanding and listening effort task with older adult hearing aid users. The DNN-listening program, which combined deep neural network-driven noise management and directional microphone processing, led to lower oxygenation in the left lateral prefrontal cortex compared to the standard-listening program, which aligned with improved speech understanding and reduced listening effort. Furthermore, the DNN-listening program lowered oxygenation, as did aided versus unaided listening in our previous study^18^. These findings suggest that a combination of beamforming and DNN-based noise management technology may lessen cognitive resource demands during listening compared to other hearing aid programs in difficult listening situations. However, the observed brain-behaviour relationships indicate that listening effort may interact with other cognitive process in complex ways, potentially influencing prefrontal engagement. Future research should explore how cognitive processes like listening effort and attention interact such as in a dual-task paradigm under varying listening conditions. Furthermore, the specific contribution of the DNN noise management feature cannot be disentangled from that of directional processing. Therefore, future work should look to isolate the individual contributions of each of these factors to better understanding their relative impact on listening effort and accuracy. Overall, these study findings support the use of fNIRS as a tool that is sensitive to differences between hearing aid features and that has sufficient spatial acuity to discriminate activity between different areas of the prefrontal cortex.

## Data Availability

The datasets generated and analyzed during the current study are available in the Open Science Framework repository at https://osf.io/57sxz/. For further information regarding data availability, please contact the corresponding author J.V.
